# Contribution of the laboratory to a diagnosis process by sequential reflective testing: Paraprotein interference on a direct bilirubin assay

**DOI:** 10.11613/BM.2021.020801

**Published:** 2021-04-15

**Authors:** Niyazi Samet Yilmaz, Bayram Sen, Ozlem Gulbahar

**Affiliations:** 1 Department of Medical Biochemistry, Polatli Duatepe State Hospital, Ankara, Turkey; 2 Department of Medical Biochemistry, Recep Tayyip Erdogan University Research and Training Hospital, Rize, Turkey; 3 Department of Medical Biochemistry, Gazi University Faculty of Medicine, Ankara, Turkey

**Keywords:** direct bilirubin, interference, paraprotein, reflective testing

## Abstract

Errors in laboratory medicine occur in the preanalytical, analytical, and postanalytical phases. The errors are mostly detected in the preanalytical period. However, analytical errors are still an important source of error, despite their frequency is reduced significantly in years thanks to developments in laboratories. In this case, an analytical error was noticed during the verification of a patient’s results. The direct bilirubin of a 66-year-old male patient admitted to the emergency department was higher than the total bilirubin. The patient’s symptoms were fatigue and dyspnoea. Albumin and haemoglobin (Hb) concentrations of the patient were significantly low. After considering the patient’s demographics and laboratory results, the laboratory specialist suspected a paraproteinemia interference. Total protein was performed as a reflective test. The albumin/globulin ratio was reversed. Thereafter, serum protein electrophoresis (SPEP) and immunofixation electrophoresis (IFE) were performed as another reflective tests, respectively. SPEP and IFE results were in favour of monoclonal gammopathy. The patient was directed to a haematologist, underwent a bone marrow biopsy, and the result was reported as Waldenstrom’s macroglobulinemia with plasma cell differentiation expressing IgM-Kappa. The patient went on a chemotherapy protocol, and his condition has been improved in subsequent months. Detection of analytical errors is of great importance, like in our case, and may be used as a tool to identify patients who have not yet been diagnosed. The laboratory specialist must dominate the entire process of each test in the laboratory, be aware of the limitations of tests, and turn these disadvantages into advantages when necessary.

## Introduction

Analytical errors have significantly been reduced with factors such as automation of analysers, reagent performance (the majority of reagents are ready to use), and participation in internal quality control and external quality assurance/proficiency testing. However, each sample can present a specific matrix that may cause irregular (individual) analytical errors (*1*, *2*). Interferences may cause such individual analytical errors and spurious results.

Paraprotein interferences have been observed on various analytical instruments and many assays/methods such as turbidimetric, nephelometric, and spectrophotometric. Immunoassays can also be affected by paraproteins, though less frequently (*3*). In previous studies, spurious results caused by paraprotein interferences have been seen on several analytes such as enzymes, electrolytes, metabolites, proteins, hormones, cardiac markers, tumour markers, and therapeutic drug monitoring analyses (*4*). Analytes such as total bilirubin, direct bilirubin, uric acid, inorganic phosphate, sodium, creatinine, C-reactive protein (CRP), high-density lipoprotein (HDL) cholesterol, vancomycin, and many other parameters were reported to be affected by paraproteinemias (*3*-*23*).

Reflective testing is the addition of new tests and/or comments to the patient’s original request by the laboratory specialist after evaluating the patient’s demographics, clinical information in the test request, and the patient’s current results (*24*-*26*). By conducting reflective tests, the laboratory specialist can make recommendations to the patient and clinician’s advantage, contribute to the diagnosis, and prevent unnecessary procedures and interventions. In this case, after revealing an analytical interference caused by paraproteinemia, the laboratory’s contribution of a patient’s diagnosis process via sequential reflective testing has been explained.

## Case report

In the postanalytical phase, during verification of patient results, it was noticed that a 66-year-old male patient’s direct bilirubin (DBIL) was higher than total bilirubin (TBIL). Blood urea nitrogen (BUN), creatinine, aspartate aminotransferase (AST), alanine aminotransferase (ALT), alkaline phosphatase (ALP), and serum electrolytes were in the reference range. Glucose concentration was slightly high, and albumin was 29 g/L (35 - 52). His haemoglobin (Hb) concentration was 70 g/L (130 – 169), whereas platelets were mildly elevated, and leukocyte count was normal (Table 1). The patient was referred to the emergency department, and DBIL was 14.2 μmol/L (0 – 3.4), while TBIL was measured 8.7 μmol/L (5.1 – 20.5). Serum indices (haemolysis, icterus, and lipaemia) were normal with photometric and visual assessment. Besides, there wasn’t any flag or warning on the analyser. The serum was not viscous, and no gel formation was present that may cause incorrect sample pipetting volume.

**Table 1:** Laboratory results of the patient

**Parameter (unit)**	**Result**	**Reference interval**
Haemolysis, icterus, lipaemia	Normal	NA
Glucose (mmol/L)	6.5	4.1 – 5.5
Blood urea nitrogen (mmol/L)	3.9	2.8 – 7.1
Creatinine (μmol/L)	69	59 – 103
Total bilirubin / Direct bilirubin (μmol/L)	8.7 / 14.2	5.1 - 20.5 / 0 - 3.4
1. Rerun	8.7 / - 4.6	/
2. Rerun	8.0 / - 19.3	/
1/3 diluted sample	10.3 / 4.6	/
Total Protein* (g/L)	91	66 – 83
Albumin (g/L)	29	35 – 52
Calcium (mmol/L)	2.19	2.20 – 2.65
Sodium (mmol/L)	137	136 – 146
Potassium (mmol/L)	4.3	3.5 – 5.1
Chloride (mmol/L)	101	101 – 109
**Parameter (unit)**	**Result**	**Reference interval**
AST (U/L)	13	0 – 50
ALT (U/L)	11	0 – 50
ALP (U/L)	96	30 – 120
Leukocytes (x10^9^/L)	6.3	4.5 – 13
Red blood cells (x10^12^/L)	3.66	4.5 – 5.9
Haemoglobin (g/L)	70	130 – 169
Haematocrit (L/L)	0.261	0.400 – 0.494
Platelets (x10^9^/L)	530	150 – 450
MCV (fL)	71	77 – 87
MCH (pg)	19	27 – 31
MCHC (g/L)	268	320 – 360
RDW (%)	20.4	11.5 – 14.5
ESR (mm/h)^†^	122	0 – 15
CRP (mg/L)^†^	123	0 – 5
Beta-2 microglobulin (mg/L)*	7.37	1.42 – 3.21
Serum Protein Electrophoresis*		
Albumin (%)	31.9	55.8 – 65.0
Alpha-1 (%)	5.9	2.2 – 4.6
Alpha-2 (%)	12.9	8.2 – 12.5
Beta (%)	10.3	7.2 – 14.2
Gamma (%)	39.0	11.5 – 18.6
M-protein (g/L)*	26.4	NA
Serum immunofixation electrophoresis*	Monoclonal IgM-Kappa	NA
Immunoglobulin G (g/L)*	11.1	7 – 16
Immunoglobulin A (g/L)*	0.3	0.8 – 4.5
Immunoglobulin M (g/L)*	52	0.5 – 3.0
FKLC (mg/L)*	142	3.3 – 19.4
FLLC (mg/L)*	18.3	5.7 – 26.3
AST - Aspartate aminotransferase. ALT - Alanine aminotransferase. ALP - Alkaline phosphatase. MCV - mean cell volume. MCH - mean corpuscular haemoglobin. MCHC - mean corpuscular haemoglobin concentration. RDW - red blood cell distribution width. ESR - sedimentation rate. CRP - C-reactive protein. Ig – immunoglobulin. FKLC – Free kappa light chains. FLLC – Free lambda light chains. NA - Not available. *The tests which performed as a reflective test. ^†^Results obtained from patient one week later.		

**Figure 1:**
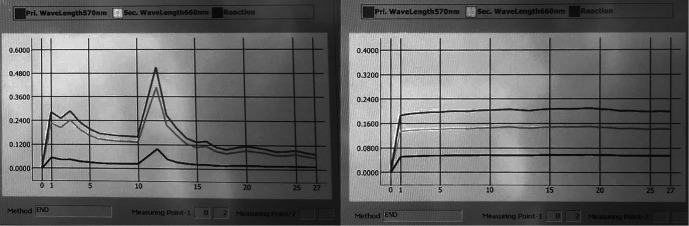
Reaction monitors of the direct bilirubin result before and after dilution.

The sample was analysed twice in a sample cup and resulted in - 4.6 and - 19.3 μmol/L for DBIL; and 8.7 and 8.0 μmol/L for TBIL. As can be seen, there was imprecision in the repeated direct bilirubin results (Table 1). The reaction monitors of the patient’s DBIL and TBIL results were examined and then compared with the other patients’ reaction monitors analysed on the same day. An unusual absorbance curve containing sharp spikes were observed in the patient’s DBIL result (Figure 1).

It was revealed from the laboratory information system (LIS) that the patient had fatigue and dyspnoea. There was not any request for radiological imaging. The patient’s diagnoses made by the doctor in the emergency department were anaemia and pain, unspecified.

Considering the patient’s age, decreased albumin level, anaemia, and the spurious direct bilirubin result, the laboratory specialist suspected a paraproteinemia, which could explain all of these findings. For eliminating suspected paraprotein interference, the serum of the patient was diluted in a 1/3 ratio. The results after dilution were TBIL = 10.3 μmol/L, DBIL = 4.6 μmol/L, and the reaction kinetics of the mentioned direct bilirubin result was normal (Figure 1), so results were verified.

All biochemistry parameters, including bilirubin concentrations, were analysed with Beckman Coulter reagents on AU680 automated chemistry analyser (Beckman Coulter, Brea, USA). Direct bilirubin (REF: OSR6211) assay in our hospital is an end-point assay. The assay was based on the formation of azobilirubin at a low pH and measured bichromatically at 570/660 nm *via* two cuvettes (colour and blank). The reagents for DBIL assay contain hydrochloric acid, sulfuric acid, and 3,5-dichlorophenyldiazonium tetrafluoroborate as the chromogen. The chromogen is added only in the colour cuvette (reaction cuvette). For measurement of DBIL, the second cuvette is used for the sample blank, and the blank cuvette’s absorbance is subtracted from the absorbance of the reaction cuvette.

In our case, peculiar spikes appeared after the “0” (*i.e.,* following the addition of the sample to the cuvette, which already contains sulfuric and hydrochloric acid and mixing stage) and “10” (following a stirring operation in cuvettes) photometric points (Figure 1). Neither diluted sample of the patient nor other patients’ samples analysed in that day did not show any spikes, and absorbance curves were parallel in these samples’ reaction data. No unusual reaction curves were observed for the patient’s other biochemistry assays.

Simultaneously, measurement of total protein was done, as a reflective test, to check the reversed albumin/globulin ratio. Due to increased total protein and reversed albumin/globulin ratio, serum was stored, and the next day another reflective test, SPEP, was performed. The doctor in the emergency department was informed that the patient may have a disease with monoclonal gammopathy and should be referred to haematology after discharge because the patient had never applied to our hospital’s haematology department before. Also, this recommendation was added to the patient’s laboratory report as a comment. A few hours later, when the patient’s file was examined, we learnt that he left the emergency department voluntarily after 1U (one unit) of red blood cell transfusion.

**Figure 2:**
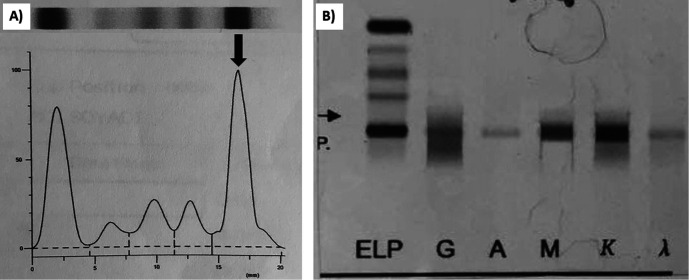
Serum protein electrophoresis and immunofixation electrophoresis results. A. The arrow assigns a monoclonal peak in the gamma region. B. A pathological band which seen in all lines.

The next day, SPEP analysis was performed in agarose gel (SAS-1 plus SAS-2, Helena Biosciences Europe, UK), and a monoclonal peak in the gamma region was detected (Figure 2). M-protein concentration was calculated as 26.4 g/L. Another reflective test, serum IFE was performed by the Interlab G26 analyser (Interlab Srl, Rome, Italy), and a pathological band was seen in all lines (Figure 2). As we saw lanes in all globulin fractions, we suspected cryoglobulinemia and/or monoclonal IgM polymerization. We could not identify the paraprotein exactly with IFE because we couldn’t repeat the analysis after treating serum with 2-mercaptoethanol (a reducing agent that breaks down disulfide bands in protein precipitates) to dissolve paraprotein precipitation (*13*, *27*). Meanwhile, nephelometric quantification revealed IgM-Kappa increase (Beckman Coulter Immage 800, Brea, USA). Although the clinician had been informed and the possible paraproteinemia mentioned as a comment in the patient’s laboratory report in previous days, for patient safety, we decided to contact the patient in the light of new results obtained.

The patient was contacted by phone and asked about his previously diagnosed diseases and medications. We learnt that the patient hadn’t had any haematological diagnosis yet. He’d been taking valsartan+thiazide, metoprolol, salicylate, clopidogrel, and metformin for hypertension, coronary artery disease, and type 2 diabetes mellitus. One week ago, the patient applied to his family doctor with fatigue, dyspnoea, and weight loss (8 kg in last year). After the family doctor noticed the patient’s Hb concentration was 78 g/L in the complete blood count, she planned urinalysis, faecal occult blood test, abdomen ultrasonography, endoscopy, and colonoscopy for suspected malignancy. The patient was kindly invited to our laboratory to give him information about his reflective test results and directed him to the haematology department. We also advised him to go on all his planned requests and procedures in case of any other malignancies. A few weeks later, the patient wanted to share his results with us. Informed consent was obtained from the patient to report these findings for scientific purposes. According to the patient’s results, the faecal occult blood test was negative, urinalysis wasn’t significant, either. Abdominal ultrasonography and colonoscopy were normal; endoscopic biopsy resulted in atrophic gastritis. Cryoglobulinemia test result was negative in another laboratory. After seen by a haematologist, the patient underwent a bone marrow biopsy. The bone marrow biopsy result was reported as Waldenstrom’s Macroglobulinemia (lymphoplasmacytic lymphoma) with plasma cell differentiation expressing IgM-Kappa. The patient went on a chemotherapy protocol, and his condition has been improved in subsequent months.

## Discussion

In this case report, the laboratory’s contribution to the diagnosis process of a patient is presented. A spurious DBIL result arose from a paraprotein interference. Thanks to the awareness of our laboratory about M protein interference, sequential reflective tests were performed. The patient underwent the biopsy process quickly and was diagnosed with Waldenstrom’s Macroglobulinemia.

It has been shown that Beckman Coulter conjugated bilirubin assay may be sensitive to paraproteinemia interference. The interference rate of the samples with monoclonal protein was found between 1.5 - 44% (*10*-*16*). The prevalence of monoclonal gammopathies in laboratories, different M-protein concentrations, and the criteria used to define the DBIL interference may be the causes of variable interference rates between studies (*12*).

Precipitation of paraproteins, sample turbidity, binding of the M-protein to the analyte or a component in the measuring system, volume displacement effect, prozone effect, hook effect, hyperviscosity and cryoglobulinemia are the mentioned mechanisms of paraproteinemia interference (*3*, *19*, *21*, *23*, *28*). However, the most common interference mechanisms are paraprotein precipitation and increased sample turbidity (*23*, *28*).

Similar to our case report, Bora and Chutia observed peculiar spikes in a patient with monoclonal gammopathy in Beckman Coulter TBIL assay (*9*). They mentioned that transient turbidity occurred in the cuvettes due to paraprotein precipitation when the serum and TBIL reagent were mixed, which led to the first peculiar spike (after the 0 point). Then, transient turbidity reappeared when the autoanalyser performed a stirring operation between “10” and “11” points, which was responsible for the second spike. We also saw peculiar spikes right after the addition of the sample in the cuvette, then following the second mixing stage.

Direct bilirubin assay carries out in a strongly acidic medium (*13*). Precipitation of paraproteins in a strongly acidic pH or mixing the constituents in the cuvettes could cause these peculiar spikes. Physicochemical properties of the M-protein, pH, ionic strength, and assay additives affect precipitation (*4*). In clinical chemistry assays, reagents include protein stabilizing agents for avoiding precipitation (*10*, *11*, *13*). The solubilisation capacity of protein stabilizing agents is probably not sufficient when protein concentrations are much higher than usual concentrations of serum proteins. The probable mechanism of interference in our case was the precipitation of excessive M-protein in a strong acid medium and resulting turbidity (*5*, *8*-*12*, *18*, *23*, *28*, *29*).

As seen in our case, paraprotein interference may cause irreproducible results in direct bilirubin assay (*10*, *12*, *13*, *15*). There may be a fluctuation pattern; the DBIL results may be negative, or higher than the TBIL results (*10*, *12*). Aggregation/precipitation suspended in a solution due to paraproteinemia can scatter light and interfere with the absorbance measurements (*30*).

For demonstrating the paraprotein interference, the assay may be performed in a test tube via manually adding reagents (*5*, *6*, *8*-*12*, *29*). The test may be analysed in a different manufacturer’s assay or another method with the same manufacturer (*20*). The parameter can also be measured with a slide-based dry technology assay (*6*, *7*, *10*-*12*). In our case, we could not perform these methods.

Removal of paraproteins by ultrafiltration or deproteinization, treatment of the sample with polyethylene glycol, and dilution of the sample are the methods recommended for eliminating paraprotein interference (*3*, *6*, *11*, *13*, *17*, *18*, *20*, *30*). Since we eliminated the interference with the dilution, we did not perform any additional procedure.

Each paraprotein is unique and may cause individual analytical errors due to interferences. Some approaches or protocols may be useful to detect these individual analytical errors systemically. Preventing the verification of negative test results or providing flags and warnings *via* LIS, interferences on several assays may be detected (*14*). Also, implementations like delta checks or consistency checks on patient results may be applied with LIS or middlewares (*15*). Implementing warning flags from instruments and reviewing the photometric reaction data can also be useful for detecting these individual analytical errors systematically (*7*, *16*, *29*). Our case is an example that such an analytical interference can be used to identify non-diagnosed patients with monoclonal gammopathy.

## Conclusion

In this case, the laboratory detected a paraproteinemia interference and contributed to the patient’s diagnosis. Communication of the laboratory specialist with the clinician and the patient has also facilitated this process. In the postanalytical phase, in the light of the laboratory’s knowledge, laboratory data were transformed for the benefit of the clinician and the patient, and future action plans for patient care were proposed.

Detection of analytical errors is of great importance, such in our case, and may be used to identify patients who have not yet been diagnosed. The laboratory specialist should be aware of the limitations of tests and turn these disadvantages into advantages when necessary.
